# A longitudinal study of bonding failure related to aspects of posttraumatic stress symptoms after childbirth among Japanese mothers

**DOI:** 10.1186/s12884-020-03099-0

**Published:** 2020-07-29

**Authors:** Yoshiko Suetsugu, Megumi Haruna, Kiyoko Kamibeppu

**Affiliations:** 1 Department of Health Sciences, Graduate School of Medical Sciences, Kyushu University, Fukuoka, Japan; 2 Department of Family Nursing, Division of Health Sciences & Nursing, Graduate School of Medicine, The University of Tokyo, 7-3-1 Hongo, Bunkyo-ku, 113-8654 Tokyo, Japan; 3 Department of Midwifery and Women’s Health, Division of Health Sciences & Nursing, Graduate School of Medicine, The University of Tokyo, Tokyo, Japan

**Keywords:** Bonding failure, Posttraumatic stress disorder after childbirth, Posttraumatic stress symptoms after childbirth, Japanese mother, Postpartum

## Abstract

**Background:**

Posttraumatic stress symptoms (PTSS) after childbirth may affect mother-infant bonding. This study examined the relationship between aspects of PTSS after childbirth and bonding failure for mothers at 1 month and 4 months after delivery.

**Methods:**

This longitudinal study surveyed 130 mothers at 1 month (T1) and 4 months (T2) after delivery. We performed multiple regression analysis with the Postpartum Bonding Questionnaire (PBQ) as the dependent variable and the Impact of Event Scale-Revised (IES-R), Edinburgh Postnatal Depression Scale (EPDS), Relationship Questionnaire (RQ), Family Adaptation, Partnership, Growth, Affection, and Resolve score (F.APGAR), and demographic data as independent variables.

**Results:**

The rate of mothers with an IES-R score of ≥ 25 was 6.2% at T1 and 3.8% at T2. The IES-R and the EPDS were relevant factors for the PBQ at T1. The IES-R was not a relevant factor, but the EPDS was a relevant factor for the PBQ at T2. The IES-R at T1 was not a predictor for the PBQ at T2. The PBQ at T1 was the largest predictor for the PBQ at T2, when compared with the EPDS, F.APGAR, and dismissive attachment pattern (RQ) at T1.

**Conclusions:**

PTSS after childbirth had a strong influence on bonding failure at T1. However, the important factor affecting bonding failure was not PTSS after childbirth, but depression at T2. If PTSS after childbirth are accompanied by depression at T2, bonding failure may be affected. Bonding failure affected by PTSS after childbirth at T1 could affect bonding failure at T2. Health professionals should assess the degree of PTSS after childbirth and start to care for mothers at T1.

## Background

The development of a relationship between a mother and newborn is the central and most important psychological process of the puerperium [1]. Emotional ties of the mother to the infant develop continuously from pregnancy over the postpartum period. Although many mothers bond easily with their infants, some fail to bond [2]. A negative birth experience for mothers, such as posttraumatic stress disorder (PTSD) after childbirth, may break the continuous development of the mother-infant bond.

PTSD after childbirth is inferred by posttraumatic stress symptoms (PTSS), whereby associations with PTSD lead to intrusions, avoidance, negative alterations in cognition and mood, and alterations in arousal and reactions [3]. The prevalence of PTSD after childbirth has been reported to be 2-14.3% at 1 to 2 weeks after delivery [4, 5], 1–8% at 4 to 6 weeks after delivery [6–13], 2.4% at 3 to 6 months after delivery [14, 15], and 1.7-3% at 1 year after delivery [16, 17]. In Japan, few reports have examined PTSD in mothers after childbirth. One study reported that the proportion of mothers with PTSD after childbirth was 13.2% among mothers of babies in the neonatal intensive care unit and 5.1% among mothers of healthy babies at 1 month after delivery [18].

A large volume of research has examined risk factors for PTSD after childbirth. Previous studies with non-Japanese women have reported that the following were associated with PTSD after childbirth: insecure attachment style [19]; previous mental health difficulties [20]; trait anxiety [20]; sense of coherence [21]; history of sexual trauma [7, 13]; previous traumatic experiences [22, 23]; maternal occupation [12]; low income [7]; depression in early pregnancy [9]; first time mother [5]; fear of birth [9]; pregnancy complications [24]; assisted vaginal birth [20]; mode of delivery [25]; emergency Caesarean section [5, 24]; Caesarean section [7, 20]; manual removal of the placenta [24]; obstetric intervention during childbirth [6]; length of labor [25]; pain during labor [7]; psychological difficulties in pregnancy [22]; low perceived control and powerlessness during childbirth [7, 21, 25]; low 5-minute Apgar score [25]; low birth weight of the infant [26]; perception of inadequate intrapartum care [6]; low levels of support from partner and staff [22]; and lack of social support [7].

PTSD after childbirth can have a negative impact on maternal feelings. Qualitative studies have demonstrated that the impacts of PTSD after childbirth affect the mother-infant relationship. In studies conducted in New Zealand, the United States, Australia, and the United Kingdom, women who experienced PTSD after childbirth reported weak maternal feelings and negative feelings and emotions toward the baby [27]; in Iran and Europe, such women reported having difficulties establishing a relationship with their infant or being unable to fulfill their duty as a mother [28, 29].

Mother-infant bonding failure can cause inappropriate rearing that leads to child abuse. In fact, in Japan, the number of cases of child abuse has been increasing annually, reaching a total of 133,778 cases in 2017 [30]. Although the rate of child abuse in the population under the age of 5 is about 1% [30], it is increasing each year. One relevant factor related to difficulties in mother-infant bonding is postpartum depression [31, 32]. Another factor that impacts bonding may be PTSD after childbirth. PTSS after childbirth could affect mother-infant bonding depending on whether there was a diagnosis of PTSD or not. It is possible that PTSS after childbirth may lead to sustained bonding failure. Although qualitative research has reported on the relevance between PTSD after childbirth and the relationship of mothers to infants, few studies have shown statistically significant findings. The purpose of this study was to examine the relationship between PTSS after childbirth and bonding failure for Japanese mothers at 1 month and 4 months after delivery, and to examine whether PTSS after childbirth could be a predictor of bonding failure at 4 months after delivery.

## Methods

### Study design and participants

This study was a prospective longitudinal study to survey healthy mothers and infants from 1 month to 4 months after delivery. Between July 2013 and April 2014, 200 mothers who had given birth at local obstetric hospitals in urban areas in Kyushu, Japan, and were undergoing a health check-up 1 month after delivery were recruited for this study.

The Research Ethics Committee of the Faculty of Medicine of the University of Tokyo, and the Kyushu University Institutional Review Board for Clinical Research approved the study, and written informed consent was obtained from all participating mothers. Inclusion criteria were women > 20 years old and infants with a normal gestational period and a birth weight of 2500–4000 g. Women who gave birth to twins or had medical problems, or who had experienced a traumatic life event, such as the death of a close relative, within 6 months were excluded. Questionnaires were completed at 1 month (T1) and 4 months (T2) after delivery at participating obstetrics clinics in a space where privacy was assured, or at home, and returned to the researcher anonymously.

#### Measurements

### Dependent variables

#### Postpartum Bonding Questionnaire

In the present study, we defined “bonding failure” as maternal problems with building bonds with their infants that contributed to problems with the mother-infant relationship. “Bonding failure” was measured by the Postpartum Bonding Questionnaire (PBQ) at T1 and T2. The PBQ was developed by Brockington [33, 34] to screen for bonding disorders, and the Japanese version of the PBQ was developed by Suetsugu et al. [35]. The validity and reliability of the scale were examined by comparing the results of mothers with and without postpartum depression. The internal consistency of the Japanese version of the PBQ was 0.84 at 1 month after delivery. The PBQ consists of four subscales: “impaired bonding” (12 items), “rejection and anger” (seven items), “anxiety about care” (four items), and “risk of abuse” (two items). Each item is rated on a 6-point scale ranging from “always” (5) to “never” (0), reflecting failure with mother-to-infant bonding in the previous week. When the statement reflects a positive emotion or attitude, the scoring is reversed. Higher scores reflect difficult mother-to-infant bonding. Each subscale independently reflected one concept of bonding failure. The total score of the PBQ shows the general tendency for bonding failure. The internal consistency of the PBQ in the present study was 0.88 (Cronbach’s alpha).

### Independent variables

#### Impact of Event Scale-Revised

In the present study, we defined “PTSS after childbirth” as a state of having PTSS after giving birth, which was assessed as the degree of symptoms determined by the Impact of Event Scale-Revised (IES-R) [36], regardless of diagnosis of PTSD. The Japanese version of the IES-R was developed by Asukai [37], and the validity and reliability of the scale were examined by comparing the results of people who had been affected by a disaster with those of the general population; internal consistency was 0.84.

The IES-R items consist of three dimensions (eight intrusion items, eight avoidance items, and six hyperarousal items) to categorize PTSS. It consists of 22 statements graded on a 5-point scale ranging from “always” (4) to “never” (0) in terms of response to a specific stressful life event for the previous week. We added the explanatory note, “Please respond based on your birth experience”.

The total score on the IES-R score was determined at T1 and T2. Higher scores reflect more PTSS. The threshold for detecting a probable diagnosis of PTSD is IES-R score ≥ 25 [36, 37]. The validity and reliability of the IES-R are well established [36, 37]. The internal consistency of the IES-R in the present study was 0.94 (Cronbach’s alpha).

### Edinburgh Postnatal Depression Scale

Depression was measured by the Edinburgh Postnatal Depression scale (EPDS) [38] at T1 and T2. The Japanese version of the EPDS was developed by Okano [39], and the validity and reliability of the scale were examined by comparing the results of mothers with and without postnatal depression at 1 and 3 months after delivery, and those of mothers with and without postnatal depression 2 years after delivery; internal consistency was 0.67/0.74, and 0.78, respectively. The EPDS is an established instrument used to screen for depressive symptoms focusing on the cognitive and affective features of depression rather than on somatic symptoms in postnatal mothers. The EPDS comprises 10 items with responses rated on a 4-point scale ranging from 3 to 0 for depressive symptoms for the previous week. Higher scores reflect more depressive symptoms. We adopted a threshold of a score > 9 for detecting a probable diagnosis of depression, because Yamashita et al. [40] suggested that Japanese mothers tend to report lower scores compared with European mothers. The internal consistency of the EPDS in the present study was 0.82 (Cronbach’s alpha).

### Relationship Questionnaire

Each mother’s attachment pattern was measured using the Relationship Questionnaire (RQ) [41] at T1. The Japanese version of the RQ was developed by Kato [42], and the validity and reliability were evaluated based on the responses of a group of university students. The RQ consists of four short paragraphs describing a secure, fearful, preoccupied, and dismissive attachment pattern. Participants rated their degree of agreement to each attachment pattern on a 7-point scale. Higher scores reflect greater recognition of themselves matching each type of relationship. The RQ was used to examine the relationship between mothers’ attachment patterns and bonding with their babies.

### Family APGAR

Family function was measured by the Family Adaptation, Partnership, Growth, Affection, and Resolve score (F.APGAR) [43] at T1. The F.APGAR was developed as a multidimensional measure of global family functioning, assessing adaptation, partnership, growth, affection, and resolve. The Japanese version was developed by Kokubu [44], and the validity and reliability were examined with participants from the general population in Japan; internal consistency was 0.86. Respondents rated their satisfaction with each of the five areas from 0 to 2 points. Higher scores reflect more satisfaction with their family function. The internal consistency of the F.APGAR in the present study was 0.91 (Cronbach’s alpha).

### Demographic data

Sociodemographic data such as age, marital status, subjective economic status, educational level, and employment status were requested on the questionnaire at T1. Obstetrical data such as history of delivery, infertility treatment, delivery method, bleeding amount, breastfeeding, gestational age, and birth weight were obtained from hospital journals.

### Statistical analysis

We compared the PBQ scores at T1 and T2 by group (i.e., the probable diagnosis of PTSD group [IES-R ≥ 25] and the normal group [IES-R < 25], using Cohen’s d. Then, multiple regression analysis was performed. The total score, and the score for each subscale of the PBQ at T1 and T2 were used as dependent variables. In the analysis of predictors, we used the PBQ score at T2 as the dependent variable and the PBQ score at T1 as one of the independent variables. Demographic data and obstetrical data, which had a small effect on the dependent variables, were deducted by use of simple regression analysis and relationships between independent variables. Although a relationship between PTSS after childbirth (IES-R) and depression (EPDS) was reported by the present research (*r* = 0.514 *p* < 0.001 at T1, *r* = 0.506 *p* < 0.001 at T2) and previous studies [45–47], the Variance Inflation Factor (VIF) value of the EPDS was low (VIF = 2.112); thus, we used both variables as independent variables. The respective standardized partial regression coefficient (B) and the coefficient of determination (R^2^) were calculated.

All analyses were performed using SPSS software, version 21.0J (IBM Corporation, New York, NY, USA), and *p* < 0.05 was considered statistically significant.

## Results

Of the 200 mothers recruited, 190 (95%) agreed to participate in this study. A total of 136 mothers returned questionnaires, and 130 (65%) questionnaires were fully completed at both 1 and 4 months after delivery. We used data from participants who returned questionnaires at both time points (*n* = 130) for evaluation. Participants’ characteristics are shown in Table 1.

**Table 1:** Characteristics of participants (*N* = 130)

Age, y		31.1 ± 4.2	
Experience of birth, n (%)	Primiparous	45 (34.6)	
	Multiparous	85 (65.4)	
Number of children, n		1.9 ± 0.8	
Gestational age, months		38.9 ± 1.1	
Birth weight, g		3046 ± 333	
Bleeding, ml		331 ± 19	
Marital status, n (%)	Married	128 (98.5)	
	Divorced	2 (1.5)	
Subjective economic status, n (%)	High	10 (7.7)	115 (88.5)
	Relatively high	58 (44.6)	
	Relatively low	47 (36.2)	
	Low	15 (11.5)	15 (11.5)
Years of education, n (%)	> 12 years	85 (65.4)	45 (34.6)
	Tertiary	39 (30.0)	
	Secondary	6 (4.6)	85 (65.4)
Employment, n (%)	Full-time	29 (22.3)	86 (66.2)
	Part-time	15 (11.5)	44 (33.8)
	Unemployed	86 (66.2)	
Attachment pattern, n (%)	Secure	60 (46.2)	
	Dismissive	9 (6.9)	
	Preoccupied	25 (19.2)	
	Fearful	36 (27.7)	
Infertility treatment, n (%)	With treatment	14 (10.8)	
	Without treatment	116 (89.2)	
Delivery style, n (%)	Vaginal birth	114 (87.7)	114 (87.7)
	Planned C/S	9 (6.9)	16 (12.3)
	Emergency C/S	7 (5.4)	
Nutrition, n (%)	Breast-feeding	78 (60.0)	78 (60.0)
	Mixed	51 (39.2)	52 (40.0)
	Milk	1 (0.8)	

C/S: caesarean section

### Rate of mothers with IES-R ≥ 25

The number of mothers with IES-R ≥ 25 was eight (6.2%) at T1 and five (3.8%) at T2 (Table 2). The total PBQ scores at T1 differed between mothers with IES-R ≥ 25 and other mothers except for “rejection and anger” at T1.

**Table 2:** Difference of PBQ between mothers with IES-R ≥ 25 and other mothers at T1 and T2 (*N* = 130)

PBQ					
	Total score (Mean ± SD)	Impaired bonding (Mean ± SD)	Rejection and anger (Mean ± SD)	Anxiety about care (Mean ± SD)	Risk of abuse (Mean ± SD)
T1					
IES-R (≥ 25), *n* = 08	24.25 ± 13.594	13.25 ± 7.421	1.88 ± 3.091	5.75 ± 2.493	0.13 ± 0.354
IES-R (< 5), *n* = 122	9.96 ± 7.758	5.03 ± 4.072	1.75 ± 2.847	2.90 ± 2.323	0.01 ± 0.091
Cohen’s *d*^*a*^	1.76	1.92	0.05	1.23	1.00
	Huge effect	Huge effect	Negligible effect	Very large effect	Large effect
T2					
IES-R (≥ 25), *n* = 05	21.60 ± 3.782	10.40 ± 1.517	3.00 ± 2.739	5.00 ± 1.225	0.40 ± 0.548
IES-R (< 25), *n* = 125	9.23 ± 8.321	4.97 ± 4.589	1.42 ± 2.622	2.31 ± 2.088	0.08 ± 0.393
Cohen’s *d*^*a*^	1.52	1.21	0.61	1.31	0.81
	Huge effect	Very large effect	Medium effect	Very large effect	Large effect

*PBQ* Postpartum Bonding Questionnaire, *PTSD* Posttraumatic stress disorder, *IES-R* Impact of Event Scale Revised, *T1* 1 month after delivery, *T2* 4 months after delivery, *SD* standard deviation.

^a^Huge effect: d ≥ 1.45, Very large effect: 1.10 < d ≤ 1.45, Large effect: 0.75 ≤ d < 1, Medium effect: 0.4 ≤ d < 0.75,

Small effect: 0.15≤ d < 0.4, Negligible effect: -0.15 ≤ d < 0.15.

### Relevant factors for bonding failure at T1

Results of the multiple regression analysis are shown in Table 3. In the analysis of the PBQ total score, “impaired bonding,” and “anxiety about care,” the IES-R (β = 0.417, *p* < 0.001; β = 0.465, *p* < 0.001; β = 0.212, *p* < 0.01, respectively) and the EPDS (β = 0.231, *p* < 0.01; β = 0.231, *p* < 0.01; β = 0.280, *p* < 0.01, respectively) were relevant factors for the PBQ. The coefficients of determination R^2^ were 0.655, 0.631, and 0.613, respectively. The multiple regression model with the dependent variables “rejection and anger” and “risk of abuse” was not satisfied.

**Table 3:** Factors relevant to bonding failure at T1 (*N* = 130)

PBQ (T1)					
	Total score	Impaired bonding	Rejection and anger	Anxiety about care	Risk of abuse
Marital status	-0.006	-0.035	-0.052	0.033	-0.008
Economic status	-0.116^*^	-0.101	0.091	-0.163^**^	-0.008
Years of education	0.090	0.028	-0.082	0.199^**^	-0.182
Employment	0.151^*^	0.140^*^	0.047	0.141^*^	-0.052
Experience of birth	-0.168^**^	-0.144^*^	-0.048	-0.304^***^	-0.059
Nutrition	-0.008	-0.017	0.047	0.022	-0.012
F.APGAR	-0.015	0.011	-0.053	-0.100	-0.005
RQ Secure	-0.216^*^	-0.191^**^	-0.068	-0.211^**^	-0.220^*^
RQ Dismissive	-0.015	-0.058	-0.169	0.020	-0.014
RQ Preoccupied	0.161^*^	0.190^**^	0.129	0.082	-0.010
RQ Fearful	0.006	-0.021	-0.023	-0.047	-0.101
EPDS (T1)	0.231^**^	0.231^**^	-0.008	0.280^**^	0.005
IES-R (T1)	0.417^***^	0.465^***^	0.180	0.212^**^	0.216
R^^2^	0.616	0.589	-0.013	0.569	0.024
*F*	16.945^***^	15.229^***^	0.868	14.107^***^	1.245

Multiple regression analysis

***p < 0.001 **p < 0.01 *p < 0.05

*T1* 1 month after delivery, *β* standardized partial regression coefficient, *R^*^*R^2*^squared multiple correlation coefficient adjusted for the degrees of freedom, *PBQ* Postpartum Bonding Questionnaire, *F.APGAR* Family Adaptation, Partnership, Growth, Affection, and Resolve, *RQ* Relationship Questionnaire, *EPDS* Edinburgh Postpartum Depression Scale, *IES-R* Impact of Event Scale Revised

### Relevant factors for bonding failure at T2

Results of the multiple regression analysis are shown in Table 4. In the analysis of the total score of PBQ, “impaired bonding,” and “anxiety about care,” the IES-R score was not a relevant factor for the PBQ, and the EPDS score was a relevant factor (β = 0.426, *p* < 0.001; β = 0.450, *p* < 0.0 1; β = 0.312, *p* < 0.01, respectively). The coefficients of determination *R*^2^ were 0.492, 0.426, and 0.371, respectively. The multiple regression model with the dependent variable “rejection and anger” and “risk of abuse” was not satisfied.

**Table 4:** Factors relevant to bonding failure at T2 (*N* = 130)

PBQ (T2)					
	Total score	Impaired bonding	Rejection and anger	Anxiety about care	Risk of abuse
Marital status	0.049	0.028	0.033	0.108	-0.023
Economic status	-0.094	-0.070	-0.052	-0.154^*^	-0.058
Years of education	-0.004	-0.012	0.031	0.113	-0.173
Employment	0.020	0.023	-0.061	-0.022	0.104
Experience of birth	-0.131^*^	-0.110	-0.052	-0.259^***^	0.025
Nutrition	-0.132	-0.077	-0.065	-0.073	-0.155
F.APGAR	-0.116	-0.105	-0.252	-0.182^*^	0.044
RQ Secure	-0.152^*^	-0.132	-0.190	-0.163	-0.075
RQ Dismissive	0.121	0.145	-0.098	-0.028	0.045
RQ Preoccupied	0.100	0.068	0.065	0.053	-0.055
RQ Fearful	-0.064	-0.079	-0.098	0.041	-0.122
EPDS (T2)	0.426^***^	0.450^***^	0.086	0.312^**^	0.154
IES-R (T2)	0.184^*^	0.133	0.093	0.110	0.215
R^^2^	0.492	0.426	0.052	0.371	0.040
*F*	10.617^***^	8.367^***^	1.548	6.841^***^	1.414

Multiple regression analysis

***p < 0.001 **p < 0.01 *p < 0.0

*T2* 4 months after delivery, *β* standardized partial regression coefficient, *R^* ^*2* ^squared multiple correlation coefficient adjusted for the degrees of freedom, *PBQ* Postpartum Bonding Questionnaire, *F.APGAR* Family Adaptation, Partnership, Growth, Affection, and Resolve, *RQ* Relationship Questionnaire, *EPDS* Edinburgh Postpartum Depression Scale, *IES-R* Impact of Event Scale Revised

### Predictors for bonding failure at T2

The results of the multiple regression analysis are shown in Table 5. The IES-R score at T1 was not a predictor of the PBQ at T2. The PBQ at T1 was the most important predictor for the total PBQ score and scores of “impaired bonding,” “rejection and anger,” and “anxiety about care” at T2. Only the EPDS score at T1 was a predictor of “impaired bonding” on the PBQ at T2. F.APGAR was a predictor for the total PBQ score and scores of “impaired bonding,” “rejection and anger,” and “anxiety about care” at T2, and the RQ2 subscale “dismissive” was a predictor for the total PBQ score and scores of “impaired bonding” at T2. Coefficients of determination R^2^ ranged from 0.482 to 0.537. The multiple regression model with the dependent variable “risk of abuse” was not satisfied.

**Table 5:** Predictors of bonding failure at T2 (*N* = 130)

PBQ (T2)					
	Total score	Impaired bonding	Rejection and anger	Anxiety about care	Risk of abuse
Marital status	0.083	0.077	0.069	0.114	-0.005
Economic status	-0.013	0.003	-0.094	-0.065	-0.042
Years of education	-0.016	0.008	0.084	0.041	-0.108
Employment	-0.053	-0.041	-0.089	-0.081	0.108
Experience of birth	0.044	0.067	-0.027	-0.015	0.021
Nutrition	-0.143^*^	-0.093	-0.083	-0.109	-0.125
F.APGAR	-0.172^*^	-0.167^*^	-0.245^**^	-0.165^*^	-0.021
RQ Secure	-0.074	-0.065	-0.169	-0.067	-0.059
RQ Dismissive	0.162^*^	0.194^*^	0.013	-0.031	0.118
RQ Preoccupied	0.022	-0.040	0.017	-0.008	0.005
RQ Fearful	-0.060	-0.067	-0.079	0.063	-0.072
EPDS (T1)	0.154	0.208^*^	-0.004	0.174	-0.083
IES-R (T1)	-0.023	-0.072	0.000	0.005	0.087
PBQ (T1)	0.520^***^	0.520^***^	0.537^***^	0.482^***^	0.257^**^
R^^2^	0.492	0.458	0.329	0.455	0.031
*F*	9.920^***^	8.787^***^	5.514^***^	8.689^***^	1.294

Multiple regression analysis

***p < 0.001 **p < 0.01 *p < 0.05

*T1* 1 month after delivery, *T2* 4 months after delivery, *PBQ* Postpartum Bonding Questionnaire, *β* standardized partial regression coefficient, *R^*^*2*^squared multiple correlation coefficient adjusted for the degrees of freedom, *F.APGAR* Family Adaptation, Partnership, Growth, Affection, and Resolve, *RQ* Relationship Questionnaire, *EPDS* Edinburgh Postpartum Depression Scale, *IES-R* Impact of Event Scale Revised

## Discussion

### Validity of the sample

Our results represented the actual situation of bonding failure related to traumatic birth experiences in healthy Japanese mothers with healthy babies. The proportion of subjects with EPDS total scores > 9 points (13%) was consistent with the incidence of postpartum depression in Japan (8–20%) [40]. The proportion of normal mothers with healthy infants who had a total IES-R score ≥ 25 at 1 month was 6.2%, slightly higher than has been reported in Japan [18], and the same as that reported in other countries (1–8%) [6–13, 24]. Of the IES-R positive group, about 40% had coexistent depression. PTSD occurs concomitantly with depression at a rate of 40–50% [48], which was consistent with our results.

### Relevant factors for bonding failure

In this study, the score on each subscale of the PBQ represented one aspect of bonding failure, and the total score showed a general trend of bonding failure.

At 1 month after delivery, the total score on the PBQ, that is, PTSS after childbirth, were a relevant factor for bonding failure. According to the results for “impaired bonding” on the PBQ, PTSS after childbirth was shown to have a strong influence on “impaired bonding.” These results were consistent with previous reports that mothers who had PTSS after childbirth had feelings of anger that were directed in multiple directions [28, 49] or became numb and demonstrated actual dissociation [28]. These feelings could result in “impaired bonding” for mothers to infants. In terms of “anxiety about care” of the PBQ, the EPDS and IES-R weakly affected this variable. This may be because the mother’s experience of PTSS after childbirth decreased her feelings of self-efficacy [49], and as a result, also decreased her feelings of self-efficacy regarding child rearing. This idea is consistent with a study that reported a relationship between PTSS after childbirth and parenting stress [50].

At 4 months after delivery, PTSS after childbirth were not a relevant factor for bonding failure, although the PBQ score in the probable diagnosis of PTSD group was higher than in the normal group. On the other hand, only depression was a relevant factor for bonding failure at this time point, although PTSS after childbirth correlated with depression in the present research and in previous studies [45–47]. Considering these results, the most important factor affecting bonding failure was not PTSS after childbirth, but depression at 4 months after delivery. If PTSS after childbirth are accompanied by depression, it may affect bonding failure.

### Predictors for bonding failure

Contrary to our expectations, PTSS after childbirth at 1 month after delivery did not predict bonding failure at 4 months after delivery. However, bonding failure 1 month after delivery strongly predicted bonding failure 4 months after delivery. Considering this finding, it is important to start an intervention for bonding failure within 1 month after delivery to prevent a prolonged occurrence of poor bonding.

Although it was weak, family support and a dismissive attachment pattern were negative predictors for bonding failure 4 months after delivery, so it is important to establish family support and prevent mothers from being isolated early after delivery.

### Limitations

All participants were Japanese and surveyed only at 1 month and 4 months after delivery, so our results may not apply to mothers in other cultures or in different postpartum periods. Moreover, because all variables were measured by self-reported assessments, data may not reflect objective findings. In addition, only mothers with stable health and babies without medical problems participated, so results may not apply to high-risk mothers and high-risk infants. It is possible that subjects who had depression did not participate in this study. Because PTSS after childbirth and bonding failure are difficult for subjects to address on a questionnaire, it is possible that some mothers with PTSS did not participate in this study.

### Clinical applications

Our new findings provide a point of view to support that mothers with bonding failure may have experienced PTSS after childbirth. In addition, our findings suggest that only screening for depression is not enough to examine the support needed for bonding relationships between mothers and babies. Health professionals should assess the degree of PTSS after childbirth and start to care for mothers within 1 month after delivery because it could affect bonding failure at 1 month after delivery. Providing support to mothers with bonding failure that is related to aspects of PTSS after childbirth could be effective, with an approach aimed at addressing experiences related to PTSS after childbirth as well as a direct approach addressing bonding failure 1 month after delivery. It is important to start an intervention for bonding failure within 1 month after delivery and to encourage the establishment of a family support system to prevent mothers from being isolated. Mothers who have depression with PTSS after childbirth at 4 months after delivery must be carefully observed, because depression affects bonding failure. We believe that our findings will contribute to the development of appropriate care models for mothers with bonding failure related to PTSS after childbirth.

## Conclusions

This study showed a relationship between PTSS after childbirth and bonding failure. PTSS after childbirth were a relevant factor for bonding failure at 1 month after delivery, but not at 4 months. The most important factor affecting bonding failure was not PTSS after childbirth, but depression at 4 months after delivery. Furthermore, bonding failure at 1 month after delivery tends to be sustained 4 months after delivery.

## Data Availability

The datasets generated and/or analyzed during the current study are not publicly available due to other ongoing studies, but are available from the corresponding author on reasonable request.

## Abbreviations

EPDS: Edinburgh PostnatalDepression Scale
F.APGAR: Family Adaptation,Partnership, Growth, Affection, and Resolve
IES-R: Impact of EventScale-Revised
PBQ: Postpartum BondingQuestionnaire
PTSD: Posttraumatic stressdisorder
RQ: RelationshipQuestionnaire
T1: 1 month after delivery
T2: 4 months after delivery
